# Overjet and Overbite Influence on Cyclic Masticatory Movements: A CT Study

**DOI:** 10.5402/2013/932805

**Published:** 2013-09-05

**Authors:** Ingrid Tonni, Massimo Pregarz, Giulio Ciampalini, Fulvia Costantinides, Christiane Bodin

**Affiliations:** ^1^Dental School, University of Brescia, Piazzale Spedali Civili 1, 25123 Brescia, Italy; ^2^Pederzoli Hospital, Radiology Department, Via Monte Baldo 24, 37019 Peschiera del Garda, Italy; ^3^Department of Medical Sciences, Surgery and Health, Dental School, University of Trieste, Via Alfonso Valerio 32, 34128 Trieste, Italy; ^4^The Gnatos Center in Brescia, Viale Duca degli Abruzzi 163, 25100 Brescia, Italy

## Abstract

*Aim*. To determine whether a relationship exists between the linear measurements of overjet and overbite and the interincisal space delimited by the morphology of the upper and lower incisors. *Method and Materials*. 30 subjects (age range from 14.1 to 34.8 years, with a median age of 23.5 years and sex ratio F/M: 5/10) with overjet and overbite equal to 2 mm were selected from a group of 381 individuals with a full and well-aligned dentition, no previous dental treatment, and no signs or symptoms indicative of temporomandibular disorder. Computed Tomography images of vinyl polysiloxane impressions of the 30 subjects' anterior teeth were acquired. The interincisal space was defined as Immediate Overjet Angle and was calculated on the Computed Tomography images. *Results*. Although the 30 subjects presented overlapping measures of overjet and overbite, the values of the Immediate Overjet Angles were different in a range of a minimum value of 12° and a maximum value of 54°. *Conclusion*. This study reveals that (1) only 30 (7.9%) of the 381 individuals considered have values of overjet and overbite equal to 2 mm and (2) the Immediate Overjet Angle values of the 30 subjects are not related to the values of overjet and overbite.

## 1. Introduction

The cyclic pattern of masticatory movements is regulated by a continuous interaction between [1]

- peripheral inputs generated by the mastication muscles, the temporomandibular joint, and the occlusion,
- central nervous system.

Several individual occlusal factors, which affect masticatory function influencing the cyclic pattern of masticatory movements, are described in the literature: the presence or absence of dental restorations [2], the overbite (OB) [3], the overjet (OJ) [4], the inclination of the occlusal plane [5–8], the occlusal guidance [9–13], and occlusal interferences [14–16]. Many studies show that the pattern of masticatory movements reflects the individual pattern of the occlusal guidance [9–13]. Shupe et al. [17] highlighted the relationship between the anterior guidance and muscle activity. Jemt et al. [18], Ehrlich et al. [19], and Yamashita et al. [20] confirmed the influence of the upper teeth lingual morphology and inclination on muscle activity and the chewing cycle. Kimoto et al. [21] demonstrated the role of the occlusal guidance, which is strictly dependent on the occlusal morphology, as a factor which influences the masticatory function and thus the activity of masticatory muscles. Ogawa et al. [8] added that the inclination of the occlusal guidance affects the masticatory function near the intercuspal area, whereas the masticatory function outside the intercuspal range is influenced by the inclination of the occlusal plane.

The influence of the linear widths of OJ and OB on the functionality of the stomatognathic system is evaluated in many studies in the literature. Different normal ranges of linear values for OJ and OB are defined [22–28] depending on their impact on the different physiological aspects of the stomatognathic system (Table 1). Ioannidou et al. [23], Lowe et al. [29], and Riedman and Berg [30] described an interdependence between OJ and OB and craniofacial morphology. Kessler [31] and Silness and Roynstrand [32] showed that the OJ/OB relationship affects periodontal conditions. The involvement and impact of OJ and OB on functional occlusion were demonstrated in numerous studies including those of Alexander et al. [33], Silness et al. [34], Bauer et al. [35], Pullinger and Seligman [36], and Pair et al. [37]. However the literature reports that orthodontic, surgical, and restorative treatments, which are performed in accordance with the normal range values for OJ and OB, are not always functional [26]. Furthermore the studies by Caio et al. [38], Glaros et al. [22], and John et al. [24] highlighted the lacking influence of OJ and OB on temporomandibular dysfunction.

Whereas occlusal morphology and occlusal guidance near the intercuspal area are considered determinants of the masticatory function, the influence of the linear values of OJ and OB on the masticatory movements remains unclear. The purpose of this study is to determine whether a relationship exists between the linear measurements of OJ and OB and the Immediate Overjet Angle (IÔA) [39, 40]. The IÔA, which was described by Bodin [40] and revisited by Abjean and Bodin [39], is used to describe the anatomical and functional interincisal spaces in the area entering and leaving the intercuspal position (ICP), where gliding contacts occur. IÔA is the angle calculated between two lines traced in the sagittal plane from the ICP of two incisors to 2 points 1 mm inferior to the ICP on the lingual surface of the upper incisor and the labial surface of the lower incisor.

## 2. Method and Materials

An experiment was carried out to investigate the relationship between the linear values of OJ and OB and the anatomical and functional interincisal spaces delimited by the IÔA [39, 40].

### 2.1. Sample

1350 subjects (age ranging from 10.9 to 61.7 years, with a median age of 24.9 years and a sex ratio F/M: 11/10) with a complete set of well-aligned teeth were evaluated during a period of 10 years in the gnathologic department of the dental clinic. 381 of these (age range from 11.3 to 56.7 years, with a median age of 24 years and a sex ratio F/M: 11/10) had no previous dental treatment (including orthodontic treatment) and no signs or symptoms indicative of temporomandibular disorders (TMD).

The measurement of the OJ and OB linear values was performed clinically in the latter group by the same operator using a decimeter. The clinical definition of OJ and OB (Posselt, 1968) was applied in this study. Only 30 individuals (age range from 14.1 to 34.8 years, with a median age of 23.5 years old and sex-ratio F/M: 5/10) of the 381 had linear values of OJ and OB equal to 2 mm. These subjects with the same OJ and OB were included in the study in order to investigate the relationship between the linear values of OJ and OB and the individual IÔAs (Immediate Overjet Angles). The other 351/381 subjects (92.1%) were excluded from the study.

### 2.2. Study Protocol

The study protocol was reviewed and approved by the Ethics Committee of the Medical School of the University of Brescia and consisted of

- impression taken from the anterior teeth in ICP,
- spiral Computed Tomography (CT) examination of the impressions,
- check of the linear values of OB and OJ and measurement of the individual IÔAs.

All the participants received an invitation letter to participate, they were fully informed about the nature of the investigation, and an informed consensus was obtained from each of them before the beginning of the study.

**Figure 1:**
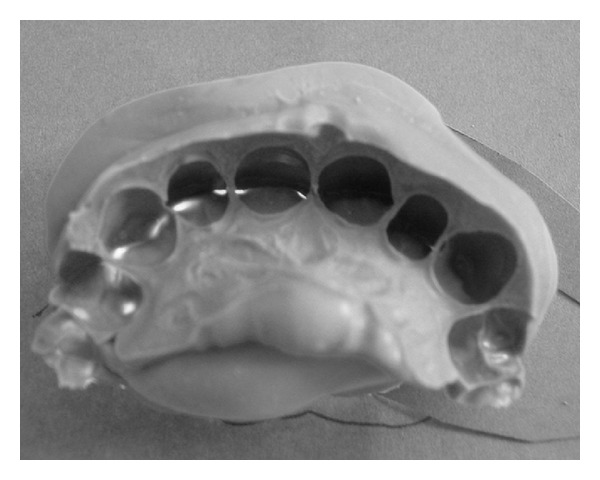
Vinyl polysiloxane impressions of the anterior teeth in ICP.

**Figure 2:**
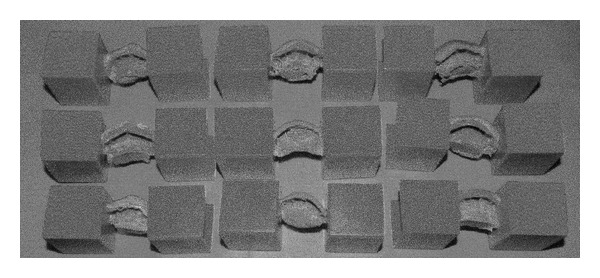
Vinyl polysiloxane impressions with the rigid plastic substrates.

An impression of the anterior upper and lower teeth was taken from each subject in the sample. They were asked to bite into a vinyl polysiloxane material Hard Putty/Fast and Light Body/Fast Express (3M ESPE Dental Products AG, Seefeld, Germany) to the ICP (Figure 1). This vinyl polysiloxane material was chosen for its property of radiopacity. Rigid plastic substrates were glued on both sides of the impressions in order to obtain stable platforms (Figure 2). The impressions, glued to their rigid substrates, were placed on the sliding table that moved into the gantry of the CT equipment as if the subject was in a supine position. Images of the vinyl polysiloxane impressions were acquired with a spiral CT avoiding irradiation of the subjects. The CT used was the multidetector computed type (CT Somatom Sensation 16, Siemens AG, Forchheim, Germany) with a 16-slice CT scanner. The high image resolution made possible the visualization of details smaller than 0.5 mm. The spiral CT scan was performed with the same intensity used to examine the internal auditory canal; the image resolution was 0.6 mm, the image acquisition was 0.3 mm, and the gap was 1 mm. 

**Figure 3:**
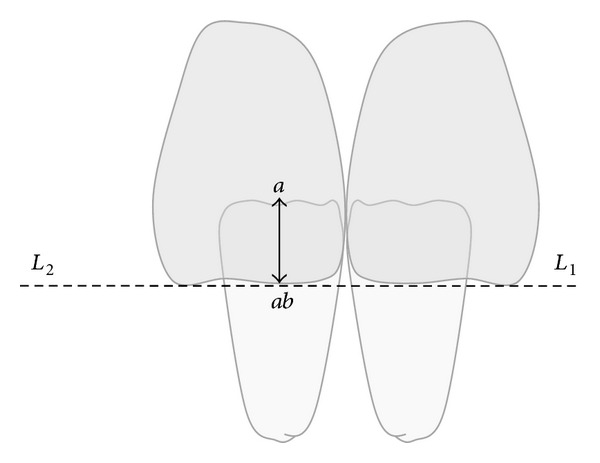
The distance *a*-*ab* represents the clinical OB.

**Figure 4:**
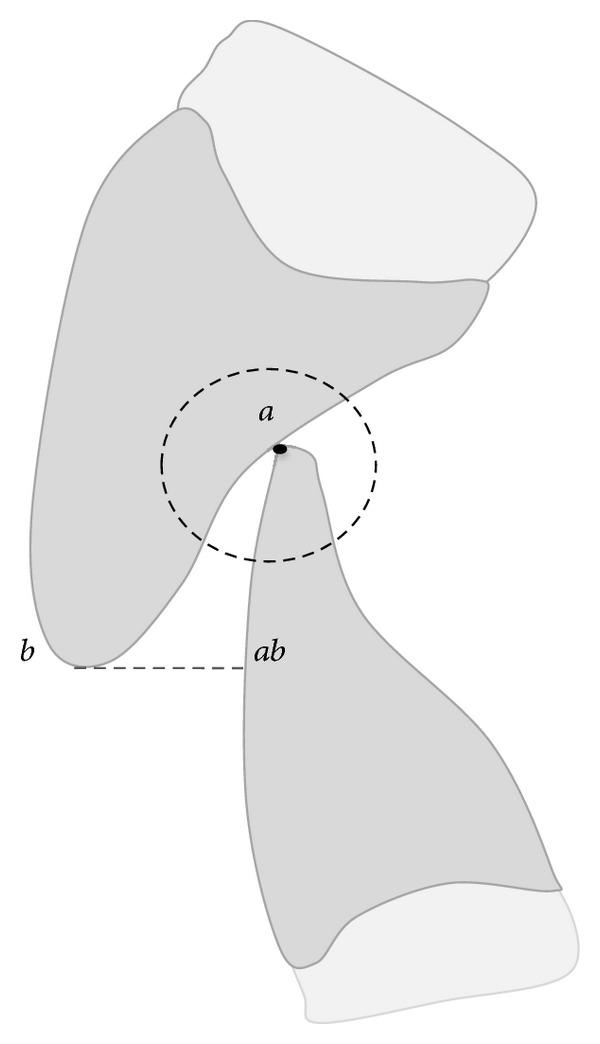
The distance *b*-*ab* represents the clinical OJ.

**Figure 5:**
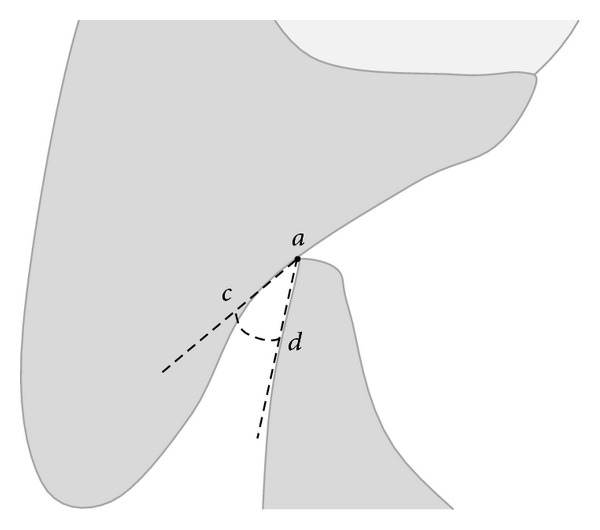
The angle bounded by the lines “*ca*” and “*ad*” is the IÔA.

**Figure 6:**
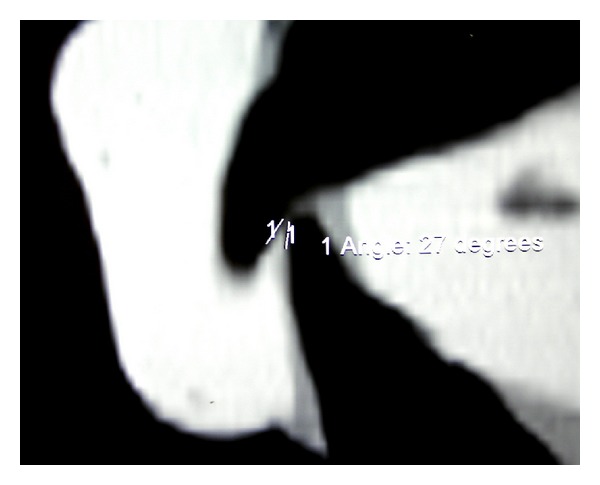
An individual IÔA calculated from CT image in the sagittal plane.

The acquired CT images were subsequently processed with the software multiplanar Reconstruction (MPR) to obtain images in the sagittal plane. The MPR software allowed orienting correctly the reconstruction plan along the axis of the section perpendicular to the incisal edge of the upper incisors (*L*_1_-*L*_2_) and passing on the most medial intercuspal point between the upper and lower central incisors (point “*a*”) (Figure 3). Point “*b*” was the corresponding point of “*a*” on the incisal margin of the upper incisor (Figure 4). From point “*a*” a line was drawn on the labial surface of the lower incisor perpendicular to the horizontal line *L*_1_-*L*_2_; the crossroad point between the 2 lines was called ab (Figures 3 and 4). The *a*-*ab* distance represented the linear value of clinical OB (Figure 3). The *b*-*ab* distance was the linear value of clinical OJ (Figure 4). This allowed verifying the linear values of OJ (2 mm) and OB (2 mm) that had been already analysed clinically. Moreover from the CT reconstruction of the interincisal relation, two points (“*c*” and “*d*”) were identified 1 mm away from the point “*a*”: the point “*c*”, located on the palatal surface of the upper incisor and the point “*d*” located on the labial surface of the lower incisor. The angle bounded by the lines “*ca*” and “*ad*” was drawn and represented the IÔA (Figure 5). This angle was calculated using the function “angle calculator” performed by the software of the Somatom Sensation 16 CT scanner (Figure 6). Reliability was assessed for the IÔA according to the intraclass correlation coefficient (ICC) that showed an excellent result (ICC = 0.92). The IÔAs were correlated with the OJ and OB linear values for all the subjects included in the study.

## 3. Results

**Figure 7:**
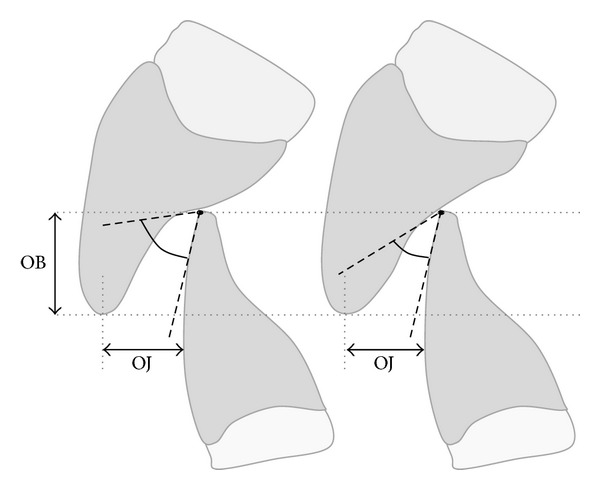
Different values of IÔAs in subjects with the same OJ and OB.

**Figure 8:**
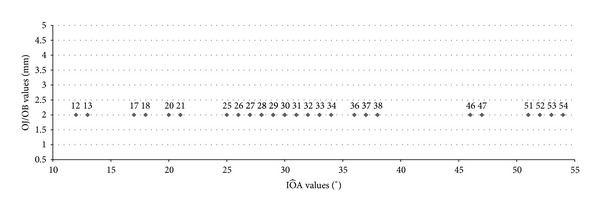
Relationship between linear values of OJ/OB and the IÔAs of the entire sample. The subjects represented in the graph are only 25 because 5 couples of individuals have the same value of IÔA. These values of IÔAs are 27°, 28°, 29°, 30°, and 37°.

30 of the 381 subjects observed had linear values of OJ and OB equal to 2 mm, which were calculated before clinically and then checked in the CT images. Although the 30 subjects studied presented overlapping measures of OJ and OB, the values of IÔA were different in a range of a minimum value of 12° and a maximum value of 54° (Figures 7 and 8).

## 4. Discussion

Linear values of OJ and OB were calculated in 381 subjects. CT images of 30 vinyl polysiloxane impressions were acquired and analysed in order to correlate linear values of OJ and OB with individual IÔAs.

Only 30 (7.9%) of 381 subjects with full dentition and without any previous dental treatment have values of OJ and OB equal to 2 mm, representing the value present in all the normal ranges for OJ and OB cited in the literature and indicating normal anatomical arrangement of the incisors and their functionality. This low prevalence suggests that linear values of OJ and OB are not common indicators of effective function in subjects with intact arches and no signs or symptoms indicative of TMD.

The 30 subjects present the same OJ and OB, but the IÔAs calculated with the software of the Somatom Sensation 16 CT scanner are different in a range from 12° to 54°. As pointed out by several authors [8, 18–21], the occlusal morphology, which is represented in this study by the anatomical aspect of the palatal surfaces of the upper incisors and the labial surfaces of lower incisors (IÔA), affects masticatory function. In the present study the linear values of OJ and OB are not correlated with the IÔAs that represent the functional space close to the ICP [39, 40]. Despite the same magnitude of OJ and OB, the subjects have a more or less wide IÔA, with a functional value dependent on the individual morphology of the incisors. The results of this investigation support the literature reports regarding the fact that dental treatments performed in accordance with the normal range values of OB and OJ are not always functional [26]. The involvement and impact of linear values of OJ and OB on the functional occlusion, as reported by some authors [33–37], need further investigation.

The IÔA is a good parameter to describe the masticatory function in the area close to the ICP [39, 40], where occlusion influence on masticatory function is greater [8]. Different from the linear values of OJ and OB, which are applied to the anterior teeth, the IÔA could also be applied to the posterior teeth in order to analyse their functional relationship. Clinically a small IÔA indicates the immediate participation of a tooth as guidance or interference, and a large IÔA indicates a delayed or lacking guidance/interference of that tooth. By visual assessment of the IÔA between the upper and lower incisors, canines, premolars, and molars, it is possible to identify a canine protected occlusion or a group occlusion and the functional sequence of teeth that work as guidance. For example, in a group occlusion, if the IÔA between UR3-LR3, UR4-LR4, and UR5-LR5 is, respectively 3°, 2°, and 1°, it indicates that the UR5, which has the smaller IÔA, results as the first tooth to guide the masticatory cycle; then UR4 and UR3 are the second, and third tooth in the guidance sequence. Furthermore for each tooth the IÔA is the “functional space in which the masticatory cycle can be accomplished” both on the anterior and the posterior teeth. Small IÔAs delimit the reduced functional space of masticatory cycles with a vertical pattern. In cases with large IÔAs the functional area of masticatory cycles is large, and the masticatory pattern is a lateral one. The clinical evaluation of the IÔA could be used both in the diagnostic approach to identify the anatomical and functional parameters of the occlusion and as an index of functional occlusion during the treatment and posttreatment periods.

## 5. Conclusions

This study reveals that

- only 30 (7.9%) of the healthy individuals with intact occlusion considered have linear values of OJ and OB of 2 mm;
- the IÔA values of these 30 individuals are not related to the linear values of OJ and OB;
- linear values of OJ and OB are not good indicators of masticatory function.

The IÔA allows detecting by clinical visual observation the interdental maxillomandibular space as an important factor of the anatomical and functional analyses of the occlusion.

## Figures and Tables

**Table 1:** Functional linear values of OB/OJ according to different authors in the literature.

Authors	Overbite	Overjet
Kinaan 1986 [25]	2 mm < OB < 4 mm	2 mm < OJ < 4 mm
Pullinger and Seligman 1991 [26]	1 mm < OB < 4 mm	1 mm < OJ < 3 mm
Glaros et al. 1992 [22]	OB < 5 mm	—
Sfondrini et al. 1997 [27]	—	OJ < 2.5 mm
Ioannidou et al. 1999 [23]	0.5 mm < OB < 4 mm	0.5 mm < OJ < 4 mm
John et al. 2002 [24]	2 mm < OB < 3 mm	2 mm < OJ < 3 mm
Svedström-Oristo et al. 2002 [28]	—	0 mm < OJ < 5 mm
